# Cross-sectional evaluation of the metabolic vulnerability index in heart failure populations

**DOI:** 10.1186/s12872-025-04758-w

**Published:** 2025-04-17

**Authors:** Kayode O. Kuku, Joseph J. Shearer, Jungnam Joo, Alan T. Remaley, Margery A. Connelly, Suzette J. Bielinski, Véronique L. Roger

**Affiliations:** 1 Heart Disease Phenomics Laboratory, Epidemiology and Community Health Branch, National Heart, Lung, and Blood Institute, National Institutes of Health, Bethesda, MD USA; 2 Office of Biostatistics Research National Heart, Lung, and Blood Institute, National Institutes, Bethesda, MD USA; 3 Lipoprotein Metabolism Laboratory, Translational Vascular Medicine Branch National Heart, Lung, and Blood Institute, National Institutes of Health, Bethesda, MD USA; 4 NMR Diagnostics Labcorp Morrisville NC, Bethesda, USA; 5 Division of Epidemiology, Department of Quantitative Health Sciences Mayo Clinic, Rochester, MN USA

**Keywords:** Heart failure, Risk score, Clinical trial, Cohort, Metabolomics

## Abstract

**Background:**

The Metabolic Vulnerability Index (MVX) is a novel multi-marker risk score derived from nuclear magnetic resonance (NMR) measures and has shown predictive value for mortality in heart failure. Hence, we aimed to evaluate the distribution of MVX and its clinical correlates within a clinical trial population and a comparable subpopulation of patients with heart failure with reduced ejection fraction and ischemic heart disease within a community cohort.

**Methods:**

We studied a clinical trial (2016–2018) and a community cohort (2003–2012), matched based on ejection fraction category and presence of ischemic heart failure. NMR LipoProfile analyses of plasma from both populations provided measures of valine, leucine, isoleucine, citrate, GlycA, and small high-density lipoprotein particles used to compute sex-specific MVX scores. Univariable and multivariable regression models assessed the relationship between MVX (modeled continuously), and selected demographic and clinical covariates.

**Results:**

Clinical trial patients (*N* = 101, median age: 63, 93% male, median EF: 28%) were younger and predominantly male compared to the cohort (*N* = 288, median age: 75, 70% male, median EF: 30%). The median MVX score was lower in the clinical trial (50, 42–61) compared to the cohort (66, 58–73). Male sex and hyperlipidemia were linked to higher MVX scores in the clinical trial, while obesity and NT-proBNP were linked to lower and higher MVX scores, respectively, in the cohort (*p* <.05). After adjusting for significant covariates from univariable analyses and age in multivariable analyses, only the associations between male sex and MVX scores in the clinical trial, and NT-proBNP levels with MVX in the cohort remained significant.

**Conclusion:**

This study highlights significant differences in MVX distribution and its clinical correlates between a clinical trial and a community cohort despite matched heart failure subtypes. These findings have important implications for interpreting and applying the score in diverse study settings.

## Introduction

Given the high burden of heart failure (HF) mortality, identifying robust risk stratification tools is crucial [1]. High-throughput molecular assays, including metabolomics, have demonstrated the potential to improve HF risk stratification [2]. Specifically, the Metabolic Vulnerability Index (MVX), a novel multi-marker score of metabolic malnutrition and inflammation derived from nuclear magnetic resonance (NMR) targeted metabolomics measurements, has demonstrated predictive value for mortality in a HF community cohort [3, 4].

Higher MVX scores have been associated with worse outcomes in observational cohorts including higher overall- and cardiovascular-related deaths [3, 5]. Despite these reports, the generalizability of MVX as a robust clinical risk assessment tool has yet to be established and requires a better understanding of its distribution and association with clinical characteristics across different study designs and HF populations.

With the aim of assessing MVX in diverse clinical settings, we conducted this study to: (1) evaluate the distribution and clinical correlates of MVX within a clinical trial population; and (2) compare the distribution and clinical characteristics associated with MVX between the clinical trial and a community HF cohort matched based on the reduced ejection fraction and ischemic heart disease criteria.

## Methods

**Fig. 1:**
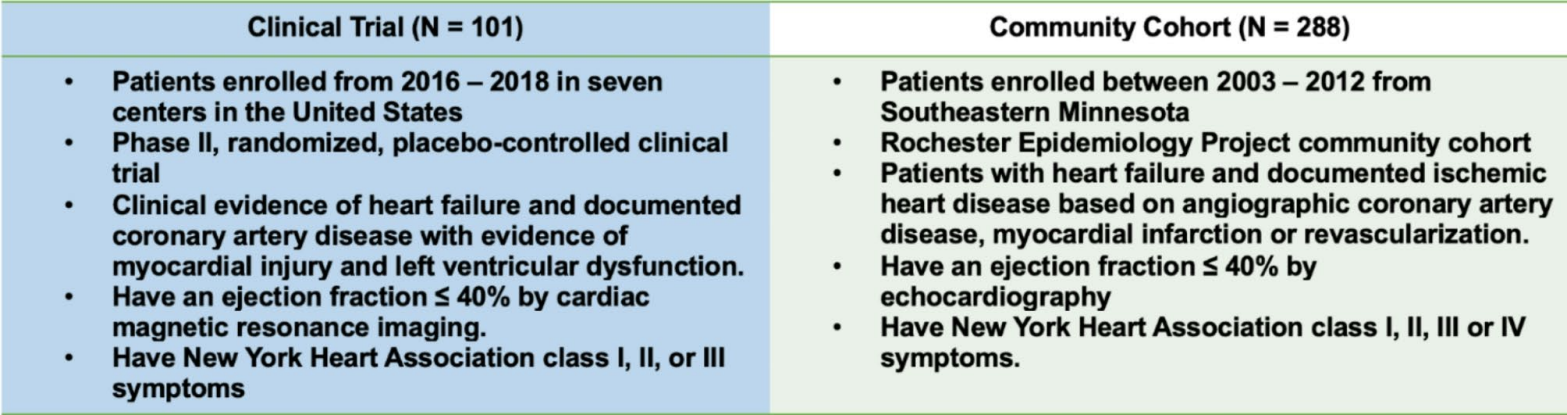
A study population: Description of the clinical trial and community cohort subset selected based on the clinical trial study criteria

The design of the clinical trial (Combination Of meseNchymal and c-kit + Cardiac stEm cells as Regenerative Therapy for Heart Failure, 2016–2018; clinicaltrials.gov Identifier: NCT02501811) which enrolled 125 patients has been previously detailed [6]. To study a subset of the cohort comparable to the clinical trial population, which included patients with ischemic HF and reduced ejection fraction (≤ 40%), we selected patients with similar profiles from the Rochester Epidemiology Project HF community cohort (2003–2012) [7] in whom the MVX had been previously assessed (see Fig. 1). Written informed consent was obtained from all participants included in both populations. Both studies adhered to relevant regulations and were approved by their respective local institutional review boards [6, 7].

NMR LipoProfile analyses of stored plasma samples collected at enrollment were conducted in both populations using the 400-MHz Vanter clinical analyzer at the National Heart, Lung, and Blood institute using the LP4 algorithm (LabCorp) [8]. Detailed information on the development of the algorithm and its association with mortality have been previously reported [3, 5]. Briefly, concentrations of valine, leucine, isoleucine, citrate, GlycA, and small high-density lipoprotein particles concentrations are determined using the NMR scan and sex-specific MVX scores are calculated using the LP4 algorithm. Sex-specific MVX scores are used in line with previous reserach to account for known sex differences in the levels of metabolite components [3, 5]. MVX scores are dimensionless and range from 1 to 100 with a higher score indicating greater metabolic vulnerability.

Clinical characteristics including hypertension, diabetes, hyperlipidemia, myocardial infarction, atrial fibrillation, and stroke were extracted from the patient medical and surgical history as part of the clinical trial [6] and in the community cohort, the same parameters were extracted from patient records by nurse abstractors [7].

The characteristics of both populations were evaluated as frequencies and percentages for categorical variables and medians and interquartile ranges for continuous variables. Regression models assessed the relationship between MVX (modeled continuously), and selected covariates based on descriptive analysis results and clinical relevance available in both populations; age, sex, obesity (body mass index ≥ 30), history of smoking, hypertension, hyperlipidemia, diabetes, cerebrovascular disease, atrial fibrillation, EF, New York Heart Association (NYHA) Class III/IV, and N-terminal pro-b-type natriuretic peptide (NT-proBNP). Age and statistically significant covariates associated with MVX (*p* <.05) in univariable models for either population were subsequently included in the multivariable analyses. Study-specific analyses were conducted throughout. Analyses were performed using R software v4.2.1 (R Core Team, Vienna, Austria).

## Results

Based on inclusion criteria and plasma availability, we studied 101 patients out of 125 in the clinical trial, and 288 patients made up the comparable cohort (based on reduced ejection fraction and ischemic heart disease) selected from the community cohort metabolomics population (*N* = 1382). Patients in the clinical trial were younger and predominantly male (median age: 63, 93% male, median ejection fraction: 28%) compared to those in the cohort (median age: 75, 70% male, median ejection fraction: 30%). The prevalence of diabetes, stroke, patients in higher NYHA classes, and NT-proBNP levels were significantly higher in the community cohort compared to the clinical trial (*p* <.05). The prevalence of cardiovascular risk factors (hypertension, hyperlipidemia, and smoking) did not differ between the two populations (*p* <.05). Table 1.

**Table 1:** Enrollment characteristics of the clinical trial and community cohort subset populations

Characteristics	Community Cohort	Clinical Trial	*p*-value
	*N* = 288	*N* = 101	
**Demographics**			
Age (years)	75 (66, 83)	63 (56, 69)	**< 0.001**
Male Sex	202 (70%)	94 (93%)	**< 0.001**
Body mass index, kg/m^2^	28(25, 32)	30 (27, 33)	**0.015**
**Medical History**			
Smoking (Ever)	203 (70%)	68 (67%)	0.600
Diabetes	122 (42%)	31 (31%)	**0.045**
Hypertension	261 (91%)	84 (84%)	0.069
Hyperlipidemia	275 (95%)	93 (93%)	0.300
Atrial Fibrillation	98 (34%)	32 (32%)	0.700
Stroke	84 (29%)	10 (9.9%)	**< 0.001**
**Clinical Presentation**			
Ejection Fraction, %	30 (24, 35)	28 (23, 32)	**0.004**
NYHA			
Class I - II	66 (23%)	86 (85%)	**< 0.001**
Class III - IV	222 (77%)	15 (15%)	**< 0.001**
NTproBNP, pg/mL	14,026 (7,209, 20,691)	347 (187, 926)	**< 0.001**

Values expressed as median (interquartile range) or n (%)

NYHA: New York Heart Association; NTproBNP: N-terminal pro-B type natriuretic peptide. Bold values indicate statistical significance

**Fig. 2:**
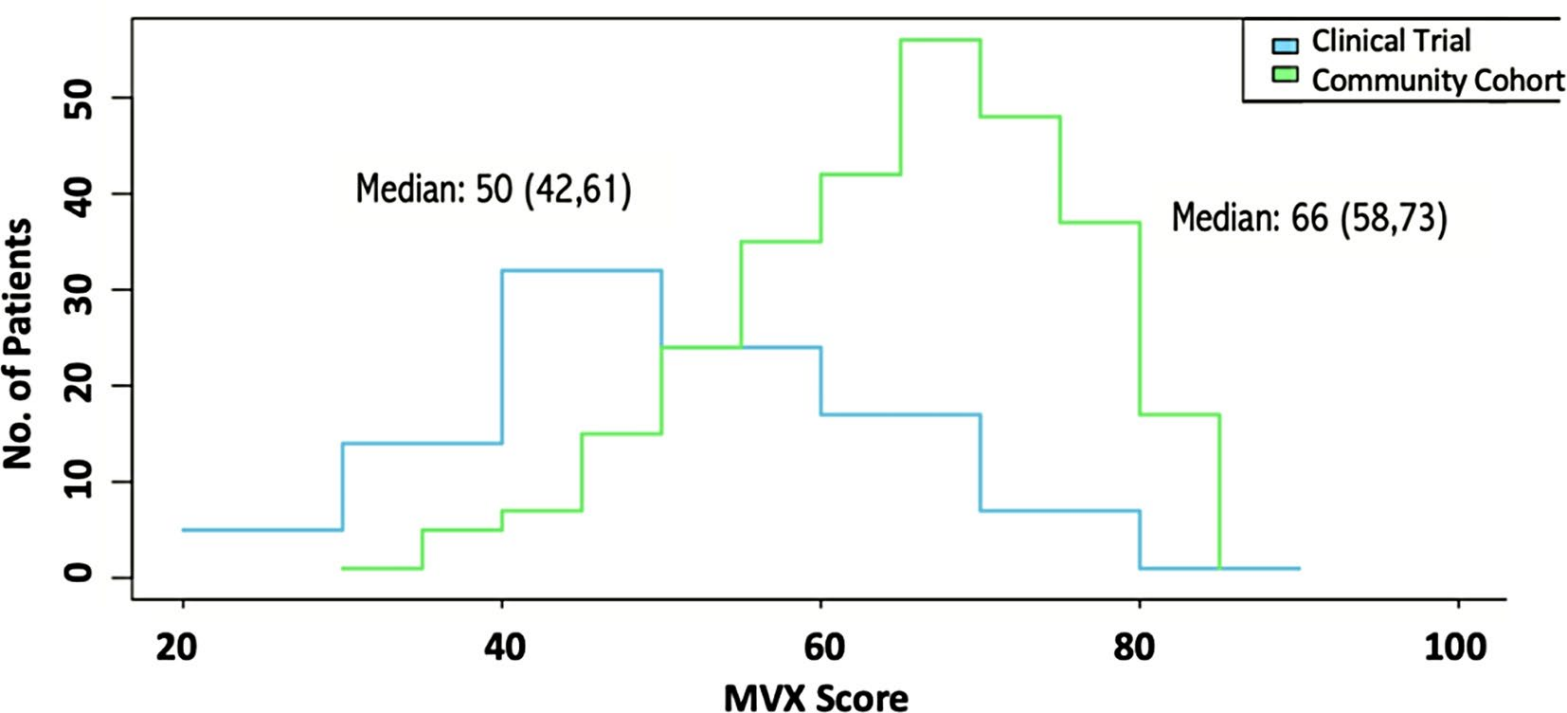
Distribution of the metabolic vulnerability index (MVX) scores in the clinical trial and cohort

The median MVX score was lower in the clinical trial (50, 42–61) compared to the community cohort (66, 58–73) Fig. 2.

In univariable analyses, male sex (β = 15.67, 95% CI: 5.99 to 25.35, *p* =.002), and hyperlipidemia (β = 9.82, 95% CI: 0.45 to 19.19, *p* =.04), were associated with higher MVX scores in the clinical trial. In the community cohort, obesity was linked to lower MVX scores (β = -3.30, 95% CI: -5.81 to -0.79, *p* =.01), while higher NT-proBNP levels were associated with elevated MVX scores (β = 4.14, 95% CI: 3.26 to 5.02, *p* <.001). After adjusting for the statistically significant covariates and age in multivariable analyses, only the positive associations between male sex and higher MVX scores in the clinical trial (β = 15.32, 95% CI: 5.14 to 25.51, *p* =.036), and NT-proBNP levels with higher MVX in the cohort (β = 4.18, 95% CI: 3.24 to 5.12, *p* <.001) remained significant Table 2.

**Table 2:** Univariable and multivariable analysis estimates represent the difference in MVX scores per unit of continuous variables or the presence (yes/no) of categorical variables

	UNIVARIABLE						MULTIVARIABLE					
	CLINICAL TRIAL			COMMUNITY COHORT			CLINICAL TRIAL			COMMUNITY COHORT		
Variable	β	95% CI	*p*-value	β	95% CI	*p*-value	β	95% CI	*p*-value	β	95% CI	*p*-value
Age	0.17	-0.12, 0.46	0.323	0.09	-0.01, 0.19	0.081	0.09	-0.19, 0.38	0.516	-0.06	-0.16, 0.04	0.266
Male sex	15.67	5.99, 25.35	**0.002**	1.04	-1.65, 3.7	0.452	15.32	5.14, 25.51	**0.036**	1.97	-0.42, 4.36	0.106
Obesity	-0.57	-5.74, 4.60	0.829	-3.30	-5.81, -0.79	**0.010**	-1.40	-6.50, 3.70	0.587	-1.30	-3.78, 1.18	0.303
Smoking	-1.11	-6.62, 4.40	0.694	1.99	-0.71, 4.69	0.150						
Hypertension	3.41	-3.47, 10.29	0.333	-1.25	-5.48, 2.98	0.564						
Hyperlipidemia	9.82	0.45, 19.19	**0.04**	-3.96	-8.80, 0.88	0.110	8.78	-0.48, 18.05	0.629	-3.15	-7.47, 1.15	0.151
Diabetes	4.32	-1.21, 9.85	0.129	-0.10	-2.59, 2.39	0.936						
Myocardial Infarction	-5.48	-17.34, 6.38	0.368	1.98	-0.55, 4.51	0.126						
Stroke	4.64	-3.96, 13.24	0.293	-2.50	-5.20, 0.20	0.070						
Atrial Fibrillation	0.07	-5.48, 5.62	0.981	1.77	-0.82, 4.36	0.183						
Ejection fraction	0.17	-0.26, 0.60	0.443	-0.11	-0.27, 0.05	0.198						
NYHA III/IV	3.61	-3.62, 10.84	0.331	2.15	-0.77, 5.07	0.150						
NTproBNP ^α^	-0.20	1.77, 1.37	0.805	4.14	3.26, 5.02	**< 0.001**	0.19	-1.43, 1.81	0.818	4.18	3.24, 5.12	**< 0.001**

Data are reported as beta coefficients (β) and 95% confidence intervals; NYHA: New York Heart Association; NTproBNP: N-terminal pro-B type natriuretic peptide (pg/ml). ^α^Log2-transformed. Significant p-values are in bold format

## Discussion

In this study, we evaluated the distribution of MVX and its associated clinical correlates in a HF clinical trial population and a comparable cohort of patients within a HF community cohort. We observed significantly higher MVX scores in the cohort compared to the clinical trial, despite similar ischemic HF and reduced ejection fraction profiles. Furthermore, the relationships between MVX and key demographic, clinical, and HF characteristics differed between the two populations. These findings imply distinct population-specific differences in the distribution and clinical correlates of MVX with ramifications for its interpretation in different clinical and community study settings.

### Distribution of MVX scores in the clinical trial and community cohorts

The median MVX score in the cohort was 66 (58, 73), similar to the median score of 65 (60, 72) observed in the broader parent HF community cohort [3]. However, despite selecting a subset of patients with similar ischemic heart disease and ejection fraction profiles as the clinical trial, MVX scores were higher than those observed in the clinical trial. Higher MVX scores in the cohort possibly reflect the broader range of comorbidities seen in a real-world HF setting [4]. In contrast, patients enrolled in clinical trials often have stringent inclusion criteria and represent a healthier HF population, hence the lower MVX scores.

### MVX and clinical correlates

No association was found between male sex and MVX scores in the cohort unlike in the clinical trial, where a positive association was observed. Given a significant association between male sex and MVX was previously observed in the community cohort [2], the absence of an association in the current cohort may be due to limited statistical power and/or differences in population characteristics. The significant association observed in the predominantly male clinical trial population however may be a reflection of the influence of sex-specific risk factors.

Multivariable analyses revealed that the associations between hyperlipidemia and MVX in the clinical trial, as well as between obesity and MVX in the community cohort, were attenuated after adjustment, indicating the influence of the other covariates. While univariable analyses initially showed a positive association between hyperlipidemia and MVX in the clinical trial, potentially consistent with its role in metabolic dysfunction [9], and a negative association for obesity defined by body-mass index in the community cohort which could imply a distinct phenotype in which excess weight is not directly linked to increased metabolic vulnerability [10]. This underscores the complex interplay of covariates influencing MVX across populations and highlights the need for further research to uncover the mechanisms linking hyperlipidemia, obesity, and metabolic vulnerability.

Notable differences across the clinical trial and community cohort populations were observed when evaluating the associations between MVX and markers of HF severity, specifically NT-proBNP levels and NYHA Class. Although the cohort had a higher prevalence of NYHA III/IV classes and higher NTproBNP levels, MVX was positively associated with NT-proBNP in this population but showed no association in the clinical trial, possibly due to differences in the patient characteristics. These findings imply that the variability in HF severity, as indicated by NT-proBNP levels [11], may impact the relevance of risk assessment tools like MVX across populations. The positive association observed between NT-proBNP and MVX in the cohort underscores the link between higher metabolic vulnerability and more advanced HF, as NT-proBNP is a recognized marker of cardiac stress and HF severity [12].

Altogether, these results highlight the value of considering heterogeneity across study designs and populations when evaluating new biomarkers and risk scores. Previous reports have shown the importance of assessing and validating established HF risk scores such as the Meta-Analysis Global Group in Chronic Heart Failure Risk Score and the Seattle Heart Failure Model in different populations [13, 14]. Evaluating risk scores across different settings enhances their generalizability and provides key insights into how they may need to be adapted for specific populations and clinical contexts to optimize predictive performance. By considering variations in disease severity and prognosis across patient cohorts, future studies can provide information to refine tools such as the MVX to improve heart failure risk assessment.

While the final role of MVX in the clinical practice remains to be fully defined, previous cohort studies have demonstrated its predictive value and its ability to capture key domains in heart failure progression —inflammation and metabolic malnutrition and its incremental predictive values over know prognostic markers in HF [3, 4, 5]. This underscores its potential clinical utility. The present study offers important insights toward its clinical adoption by showing its generalizability across populations and study designs.

The study findings should be interpreted in the context of a few limitations. First, we studied subsets of the original populations. Future study designs, with more racially and ethnically diverse patients are needed to validate these findings. Additionally, we acknowledge potential limitations from unmeasured confounding and differences in data collection methods.

This study has several notable strengths. First, we used comprehensive, high-quality data from both a rigorously controlled clinical trial and a real-world community cohort, allowing for a unique comparison between structured trial conditions and real-world clinical practice. Second, by evaluating MVX in a clinical trial and cohort, this study captured a clinically diverse spectrum of patients with HF to enhance the understanding of the novel score. Thirdly, this study highlights the value of comparing different designs to assess the generalizability of risk assessment tools specifically by examining how scores may perform differently in controlled environments versus real-world settings.

## Conclusion

While MVX shows promise as a clinical risk assessment tool in heart failure, this study reveals important differences in MVX distribution and its associations with clinical characteristics between a clinical trial population and a comparable community cohort subset. These findings underscore the need for further research to validate MVX as a robust risk tool and to explore its ability to capture unique metabolic vulnerabilities across heterogeneous HF populations.

## Data Availability

Available on reasonable request to the corresponding authors.

## Abbreviations

HF: Heart Failure
MVX: Metabolic Vulnerability Index
NMR: Nuclear Magnetic Resonance
NYHA: New York Heart Association
NT-proBNP: N-terminal pro-b-type Natriuretic Peptide
